# Select Ethical Aspects of Next-Generation Sequencing Tests for Newborn Screening and Diagnostic Evaluation of Critically Ill Newborns

**DOI:** 10.3390/ijns7040076

**Published:** 2021-11-08

**Authors:** Kuntal Sen, Jennifer Harmon, Andrea L. Gropman

**Affiliations:** 1 Division of Neurogenetics and Developmental Pediatrics, Center for Neuroscience and Behavioral Medicine, Children’s National Hospital, Washington, DC 20010, USA; ksen2@childrensnational.org; 2 Rare Disease Institute, Children’s National Hospital, Washington, DC 20010, USA; jharmon2@childrensnational.org

**Keywords:** critically ill newborns, ethics, genomic sequencing, newborn screening, policy, rapid whole exome sequencing, rapid whole genome sequencing

## Abstract

In this review, we analyze medical and select ethical aspects of the increasing use of next-generation sequencing (NGS) based tests in newborn medicine. In the last five years, there have been several studies exploring the role of rapid exome sequencing (ES) and genome sequencing (GS) in critically ill newborns. While the advantages include a high diagnostic yield with potential changes in interventions, there have been ethical dilemmas surrounding consent, information about adult-onset diseases and resolution of variants of uncertain significance. Another active area of research includes a cohort of studies funded under Newborn Sequencing in Genomic Medicine and Public Health pertaining to the use of ES and GS in newborn screening (NBS). While these techniques may allow for screening for several genetic disorders that do not have a detectable biochemical marker, the high costs and long turnaround times of these tests are barriers in their utilization as public health screening tests. Discordant results between conventional NBS and ES-based NBS, as well as challenges with consent, are other potential pitfalls of this approach. Please see the Bush, Al-Hertani and Bodamer article in this Special Issue for the broader scope and further discussion.

## 1. Introduction

With the rapid evolution of molecular genetics, next-generation sequencing (NGS) testing methods such as exome sequencing (ES) and genome sequencing (GS) are being increasingly used for diagnostic evaluation of critically ill infants as well as for newborn screening (NBS) [1,2,3]. They have been applauded for the promise they hold for the prompt and precise diagnosis of pre-symptomatic as well as sick infants. However, there are several psychosocial and ethical dilemmas surrounding the use of these tests under these circumstances that require discussion. Many of these issues have been historically associated with ES and GS, in general; however, other challenges are unique regarding their use in neonatal medicine.

## 2. Next-Generation Sequencing for Newborn Screening

In the past few years, there have been several studies by various groups to explore the use of NGS-based tests such as ES and GS for NBS. While these techniques may enable clinicians to screen for rare diseases that do not have any reliable biochemical markers, there are several moral caveats to be considered. [1,2,4].

NBS was designed as a universal cost-effective screening test aimed at early detection of treatable hereditary (and a few non-hereditary) conditions to prevent long-term mortality and morbidity [5]. The test utilizes dried blood spots from heel prick samples obtained typically on day one of life. The test screens for an array of monogenic inborn errors of metabolism (IEM), in addition to endocrine disorders, hemoglobinopathies and immunodeficiency syndromes [6]. The Advisory Committee on Hereditary Disorders in Newborns and Children (ACHDNC) makes recommendations to the individual states I the USA regarding potential new disorders to be added to the NBS. Currently, there are 35 core and 26 secondary conditions on the recommended uniform screening panel (RUSP). The American College of Medical Genetics publishes action sheets (or ACT sheets) for positive screens, to provide uniform practice guidelines for pediatricians, neonatologists and biochemical geneticists [7]. Some conditions which may present with metabolic crisis (e.g., organic acidemia) and warrant prompt evaluation in an inpatient setting, whereas others with insidious onset (e.g., phenylketonuria) are followed up in an outpatient setting.

Robert Guthrie devoted several years to developing the first screening test utilizing the bacterial inhibition assay for phenylketonuria [8]. The NBS is often referred to as ‘the PKU test,’ which remains a testimony to his career. The introduction of tandem mass spectrometry (MS/MS) revolutionized the landscape of NBS as the technique allowed screening for many disorders due to its ability to measure several analytes (plasma amino acids and the acylcarnitine profile) simultaneously [9]. The testing laboratories utilize national databases to determine the cut-off values for analytes and sometimes may involve second-tier tests. In the last decade, with the discovery of novel therapies, other disorders including storage diseases, X-linked adrenoleukodystrophy (X-ALD) and spinal muscular atrophy have been included in the RUSP for several states, which use different technologies for the screening test [10,11,12,13,14]. The literature suggests that screening for X-ALD poses a plethora of dilemmas, and there have been ongoing modifications to the follow-up protocol. The challenges include establishing contact with other family members across different generations who may be affected based on an X-linked inheritance pattern, and care co-ordination with sequential neuroimaging to identify those who will develop the cerebral form of the disease [15]. Massively parallel sequencing such as ES and GS has been used abundantly for diagnostic evaluation of pediatric genetic diseases, and clinical researchers finally decided to interrogate its potential for NBS [14]. The National Human Genome Research Institute funded a cohort of studies to evaluate the medical, economic and psychosocial effects of integrating exome sequencing (ES) for NBS, which led to the formation of Newborn Sequencing in Genomic Medicine and Public Health.

North Carolina Newborn Exome Sequencing for Universal Screening (NC NEXUS) enrolled 106 newborns and children with previously diagnosed metabolic diseases and hearing loss, and ES results confirmed an underlying diagnosis in 88% and 18% of the patients, respectively. The results included pathogenic variants in hereditary cancer syndromes in two children, and 1.8 variants per patient showing a carrier status for recessive conditions [1]. The BabySeq Project is another noteworthy study from this cohort. The group enrolled and randomized half of the families of newborns from Brigham Women’s Hospital and Boston Children’s Hospital to receive ES-based NBS. Out of 159 newborns enrolled, 15 were found to be at risk of childhood-onset conditions. Risk of adult-onset diseases, a carrier status and pharmacogenomic variants were detected in 3.5%, 88% and 5% of subjects, respectively. Testing parental samples resulted in interpretation of variants in 8% of cases [2,4].

Utilization of ES-based NBS is advantageous as it allows for screening of several monogenic conditions that do not have a consistent analyte that can be measured using MS/MS. For instance, mitochondrial disorders and proximal urea cycle disorders have historically been impossible to screen, despite the availability of therapies and interventions [16,17]. ES-based NBS could also shorten the diagnostic odysseys for those genetic diseases where there is no precise treatment, but where early diagnosis can have a significant impact on the medical care and parental perspectives. However, this rationale defies the very ethos of the Wilson and Jungner criteria, which emphasize the actionability of the disorder and cost-effectiveness of the test as the core principles of NBS [18]. The high costs and long turnaround times of NGS are obvious barriers in the current scenario, which hinder its integration as a widespread public health screening test [6]. There are also studies that suggest that the results of conventional NBS and ES-based NBS may be discordant, and the latter alone may not very specific in screening for IEM [19,20].

While shortening the diagnostic journey for infants who are sick can substantially change family’s viewpoints, diagnosis of childhood-onset conditions and carrier status can cause untold and unwarranted anxiety [1,3,4]. The BabySeq project did include an option to disclose the results of adult-onset conditions and applied a post-consent survey to better delineate parental perspectives [2]. NBS has always been an ‘opt out’ rather than an ‘opt in’ test, where families are required to actively decline it if they do not wish for their newborn to receive it. This is based on the dramatic success of the program, which has led to the slogan “newborn screening saves lives.” Parents often do not understand the purpose and process of NBS. If ES or GS were ever to become a standardized screening tool, the consenting process would need to be robust and standardized, as was proposed in the pilot studies. Reporting and resolution of variants of uncertain significance may pose several challenges for the molecular genetics laboratories as well as the clinical teams following up with these families [21]. The follow-up testing and clinic visits secondary to unclear results have been known to cause substantial financial and psychological strain for families even with the conventional NBS, and this can only be accentuated with the incorporation of ES-based NBS, unless meticulous consent and education can be ensured [2,6,22,23]. As per recommendations from the Pediatric Task Force of the Global Alliance for Genomics and Health, each jurisdiction needs to resolve ethical and policy issues regarding the disclosure of incidental or secondary findings to families, as well as the ownership, appropriate storage and sharing of genomic data. Ultimately, the best interests of children should form the basis of all decisions [21]. Since medical geneticists, genetic counsellors and metabolic dietitians are still a small group, such a drastic upgrade in NBS may also cause disruption to the access to and uniformity of care [24].

## 3. Next-Generation Sequencing for Diagnostic Evaluation of Critically Ill Newborns

While the newborn screening programs and the BabySeq project have focused on predicting and preventing future disease in pre-symptomatic infants with genetic disorders, another active area of investigation has focused on the use of rapid genomic sequencing techniques in the evaluation of critically ill infants.

Studies have estimated that genetic diseases are present in approximately 16% of neonates in regional ICUs; furthermore, there is high mortality in infants with genetic diseases, which accounts for an estimated 20% of deaths in this age group, and in one center, accounted for an estimated 45% of neonatal intensive care unit (NICU) deaths over a ten-year period [25,26]. It is proposed that the primary benefits of early genetic diagnosis in these patients include both the rapid implementation of targeted interventions that may decrease morbidity, as well as the rapid identification of likely futile intensive care in the course of what may otherwise be a protracted diagnostic approach during, which parents may experience “inappropriate hope” or “needless guilt.” Meanwhile, secondary benefits may include guiding parents regarding the risk of recurrence in future children as well as possible overall healthcare cost reductions [1,27].

Since 2015, several studies have sought to investigate the clinical utility of genomic sequencing in the NICU setting (Table 1). In these studies, the percentage of patients receiving a genetic diagnosis as a result of NGS has ranged from 21 to 57%, with results returned in a range of 2.3 to 95 days and the majority of studies utilizing rapid exome or genome sequencing and returning the results in fewer than 21 days (Table 2) [28,29,30,31,32,33,34]. Comparatively, a retrospective study comparing the diagnostic yield of genomic testing showed similar diagnostic sensitivity of rapid whole genome sequencing (rWGS), with 43% of infants receiving a genetic diagnosis compared to only 10% diagnostic sensitivity in infants who underwent a standard genetic testing protocol; similarly, another research group obtained a genetic diagnosis for 8/20 critically ill newborns using a targeted gene panel approach based on phenotypic presentation, and was able to obtain a genetic diagnosis for an additional five patients when whole exome sequencing (WES) was offered to those patients with a negative gene panel [35,36]. Interestingly, one study showed that the phenotype of neonates was a poor predictor of the underlying genotype in 90% of patients evaluated in the ICU setting with WGS, suggesting that standard genetic evaluation would likely delay the diagnosis for the majority of these critically ill patients [31]. These conclusions were supported by the results of the NSIGHT2 trial, in which a subset of patients was selected to receive ultrarapid whole genome sequencing (urWGS) as a first-tier diagnostic; in general, these patients were more unstable and had differential diagnoses that included rare disorders requiring specific targeted therapies to prevent morbidity and mortality [34]. While urWGS was more costly than the other testing modalities (rWES and rWGS) evaluated in this study, the percentage of patients that received a genetic diagnosis was significantly greater in the urWGS cohort, with a similarly greater proportion of infants in that group receiving a diagnosis for which immediate intervention was available [33].

The majority of these studies have quantified changes in management as a secondary measure and have shown repeatedly that these NGS results and subsequent genetic diagnoses have a direct impact on the decisions made by clinicians and families. These changes have encompassed both escalation in patient care (e.g., placement of a gastrotomy tube in a patient determined to have chronic feeding difficulties), as well as limitations in care (e.g., deferral of heart transplant in a patient expected to have a poor neurological prognosis) [28,30,31,32,34]. In addition to exploring the impact of rWES on acute ICU management, the most recent study reported by Freed et al. demonstrated the feasibility of implementing a rapid genomic sequencing protocol using a commercial send-out lab rather than an internal research-based testing platform, and included a full informed consent process with the option to receive secondary results [34]. As these studies have continued to consistently show a high yield of diagnoses contributing to alterations in management, and as commercial platforms have made these diagnostic modalities more accessible, a question has emerged: should rapid genomic sequencing be considered the standard of care for initial genetic diagnostic evaluation in critically ill infants? At the same time, a second question has also moved more clearly into focus: what are the ethical implications and challenges of utilizing genomic sequencing in the intensive care setting?

While the feasibility of rapid genomic sequencing for the timely diagnosis of suspected monogenic disorders in critically ill newborns has emerged only over the last decade, much accompanying literature over the same time period explores the potential technical and ethical concerns that may limit the widespread adoption of this testing approach. These include the current payer system that typically reimburses only outpatient genomic studies, the challenge of timely resolution of variants of unknown significance (VUS) and the possible impacts of rapid transition to genetic testing on parental anxiety and subsequent parent-infant bonding [27,37]. One of the most often and extensively discussed ethical concerns is the question of secondary findings, including the reporting of childhood-onset diseases not related to the patient’s clinical presentation at the time of testing, and even more fraught, the reporting of adult-onset diseases. One case report in the literature from the BabySeq project highlights the moral distress felt by study researchers upon finding a *BRCA2* mutation in a male infant whose parents had not consented to receive information regarding adult-onset disease [38]. As a result of this moral distress, the research team approached the IRB for permission to re-consent the family to receive information regarding adult-onset disease. In this case, the study protocol was changed so as to enroll only families who would consent to receiving information about adult-onset diseases, so as to avoid further ethical dilemmas and subsequent moral distress for laboratory personnel who could ostensibly know information regarding an actionable disease with no recourse to provide that information to the person it most directly affected [38]. While some researchers have argued that testing for genetic susceptibilities to adult-onset diseases is a potential violation of the future autonomy of the infant who has undergone genomic testing, others have developed ethical models that consider the interests of the family as a unit, in which the wellbeing of the infant undergoing testing is dependent on the wellbeing of the family unit as a whole [39,40]. With the specific example of this child found to have a *BRCA2* mutation, the potential impact of this autosomal, dominant adult-onset condition on the health and mortality of the child’s affected parent was considered a threat to the wellbeing of the family unit, which posed a credible downstream threat to the patient’s health as well [40,41].

Another ethical consideration is access to these technologies, which is limited by several factors, including availability of in-house testing and the associated costs of genomic testing in the setting of uncertainty regarding the likelihood of insurance reimbursement for these testing strategies. Several studies have suggested that panel-based testing could prove a reasonable alternative strategy to accomplish rapid diagnosis in the ICU setting, with the proposed benefits of ensuring that a greater number of patients in need of these rapid diagnoses receive them, while also decreasing costs. In one study, a panel of 4503 genes was utilized for rapid evaluation of critically ill neonates; in the study, this panel-based approach yielded a diagnosis in 10/20 (50%) cases and a partial diagnosis in an additional 1/20 (5%) cases, similar to those results reported in the genomic sequencing studies referenced in Table 2 [42]. Furthermore, the panel-based approach cost $6000 in this study compared to the ~$16,000–$17,500 cost reported for rWGS in other studies [42]. Despite these encouraging results suggesting possible non-inferiority of the panel-based approach, issues may arise from inconsistencies in variant detection between two modalities; for example, the GEMINI study, which directly compared the results of a sequencing panel containing 1722 genes to rWGS, demonstrated discordance between the two platforms in 27% of diagnoses [43]. While many of these were due to technical differences between the platforms, generally stemming from the known inability of the sequencing panel to detect copy number variants, a number resulted from discordance in variant analysis both with respect to the algorithms used to detect and filter variants as well as the methods used to analyze the pathogenicity of detected variants [43]. These findings demonstrate that technical factors add an additional layer of complexity into this process, necessitating an even more nuanced consideration when balancing all the factors that may influence the utility of one diagnostic strategy over another, especially regarding the potentially extensive amount of data analysis required for interpretation of genomic sequencing compared to the inherently more focused datasets resulting from panel-based approaches.

As sequencing platforms and informatic systems designed to quickly analyze large amounts of genomic data become increasingly agile, and as these testing modalities become increasingly available through commercial means, the likelihood that institutions will transition toward genomic sequencing as a first-line genetic testing strategy continues to increase. As such, our resolve to tackle and mitigate the ethical concerns that accompany these testing modalities must continue to strengthen as well.

## 4. Conclusions

This paper reviewed select ethical aspects regarding the use of ES and GS in NBS and in the diagnostic evaluation of critically ill newborns (please see the Bush, Al-Hertani and Bodamer article in this Special Issue for the broader scope and further discussion). The clinical utility and cost-effectiveness of NGS-based NBS are yet to be established. Dr. Francis Collins, NIH Director, has remarked: “*…whether you like it or not, a complete sequencing of newborns is not far away*” [44]. Genomic sequencing has caused a paradigm shift in the diagnosis of orphan diseases and will inevitably become a part of newborn screening in the near future. Yet, communities of medical geneticists, neonatologists, molecular geneticists and policymakers need to come together as a team to carefully analyze the findings of the pilot studies and improve on the shortcomings of the technique before this can become a reality. In the critically ill population, despite the potential pitfalls, multiple studies have demonstrated that both clinicians and parents have overwhelmingly positive impressions of the impact of rapid genomic sequencing in the ICU setting, regardless of whether the result returned was diagnostic [45,46,47].

## Figures and Tables

**Table 1:** Summary of studies investigating clinical feasibility and utility of NGS in critically ill neonates.

Authors	Year Published	Study Design	Patient Location	Type of Test	Medium Turnaround Time
Willig et al.	2015	Retrospective	NICU/PICU	Trio rWGS	23 days
Van Diemen et al.	2017	Prospective	NICU/PICU (Age < 1 year)	rWGS	12 days
Meng et al.	2017	Retrospective	NICU/PICU/CICU	WES (proband only/trio/rapid trio)	Proband WES: 95 days Trio WES: 51 days rWES: 13 days
French et al.	2019	Prospective	NICU/PICU	Trio rWGS	27 days
Elliott et al.	2019	Prospect	NICU	Trio rWES	7.2 days *
Kingsmore et al.	2019	RCT	NICU/PICU/CICU	urWGS/rWGS/rWES	urWGS: 2–3 days rWGS/rWES: 11.8 days
Freed et al.	2020	Prospective	NICU/PICU/CICU	Trio rWES	9 days

NICU, neonatal intensive care unit; PICU, pediatric intensive care unit; CICU, cardiac intensive care unit; WES, whole exome sequencing; WGS, whole genome sequencing; rWES, rapid whole exome sequencing; rWGS, rapid whole genome sequencing; urWGS, ultra-rapid whole genome sequencing, RCT, randomized clinical trial. * Time to preliminary results.

**Table 2:** Results of studies investigating clinical feasibility and utility of NGS in critically ill neonates.

Authors	Number of Participants	Number of Diagnoses	Number with Changes in Management	Number with Escalation of Care	Number with Limitation of Care
Willig et al.	35	rWGS 20/35 (57%)	13	6	6
Van Diemen et al.	23	7/23 (30%)	Not reported	Not reported	Not reported
Meng et al.	278	102 (36.7%) Subset: rWES 32/63 (50.8%)	53 Subset rWES: 23/32	12	19
French et al.	195	40 (21%)	12	5	7
Elliott et al.	25	18 (72%)	15	4	3
Kingsmore et al.	213	49 (24%) urWGS 11/24 (46%) rWGS 18/94 (19%) rWES 19/95 (20%)	Not reported	Not reported	Not reported
Freed et al.	46	20 (43%)	24	5	5

rWES, rapid whole exome sequencing; rWGS, rapid whole genome sequencing; urWGS, ultra-rapid whole genome sequencing.
